# RAndomized Clinical Trial Of NAfamostat Mesylate, A Potent Transmembrane Protease Serine 2 (TMPRSS2) Inhibitor, in Patients with COVID-19 Pneumonia

**DOI:** 10.3390/jcm12206618

**Published:** 2023-10-19

**Authors:** Teresa Maria Seccia, Tungalagtamir Shagjaa, Margherita Morpurgo, Brasilina Caroccia, Viola Sanga, Sonia Faoro, Francesca Venturini, Girolama Iadicicco, Sara Lococo, Maria Mazzitelli, Filippo Farnia, Paola Fioretto, Yusuke Kobayashi, Dario Gregori, Gian Paolo Rossi

**Affiliations:** 1 Internal Emergency Medicine Unit, Specialized Center for Blood Pressure Disorders-Regione Veneto, Department of Medicine—DIMED, University of Padua, 35128 Padua, Italy; teresamaria.seccia@unipd.it (T.M.S.); tungalagtamir.shagjaa@studenti.unipd.it (T.S.); sangaviola.md@gmail.com (V.S.); 2 Department of Pharmaceutical & Pharmacological Sciences, University of Padua, 35131 Padua, Italy; margherita.morpurgo@unipd.it; 3 Pharmacy, University Hospital of Padua, 35126 Padua, Italy; sonia.faoro@aopd.veneto.it (S.F.); francesca.venturini@aopd.veneto.it (F.V.); girolama.iadicicco@aopd.veneto.it (G.I.); 4 Pneumology, University Hospital of Padua, 35126 Padua, Italy; sara.lococo@aopd.veneto.it; 5 Infectious Diseases, University Hospital of Padua, 35126 Padua, Italy; maria.mazzitelli@aopd.veneto.it; 6 Internal Medicine 3, University Hospital of Padua, 35128 Padua, Italy; filippo.farnia@gmail.com (F.F.); paola.fioretto@unipd.it (P.F.); 7 Yokohama City University, Yokohama 236-0027, Japan; yusuke.kob@live.jp; 8 Biostatistics, Epidemiology and Public Health Unit, University of Padua, 35131 Padua, Italy; dario.gregori@unipd.it

**Keywords:** nafamostat mesylate, SARS-CoV-2, COVID-19, safety, transmembrane protease serine 2 TMPRSS2, coagulation

## Abstract

Even though SARS-CoV-2 was declared by WHO as constituting no longer a public health emergency, the development of effective treatments against SARS-CoV-2 infection remains a critical issue to prevent complications, particularly in fragile patients. The protease inhibitor nafamostat, currently used in Japan and Korea for pancreatitis, owing to its anticoagulant properties for disseminated intravascular coagulation (DIC), is appealing for the treatment of COVID-19 infection, because it potently inhibits the transmembrane protease serine 2 (TMPRSS2) that, after virus binding to ACE-2, allows virus entry into the cells and replication. Moreover, it could prevent the DIC and pulmonary embolism frequently associated with COVID-19 infection. The goal of the RAndomized Clinical Trial Of NAfamostat (RACONA) study, designed as a prospective randomized, double-blind placebo-controlled clinical trial, was to investigate the efficacy and safety of nafamostat mesylate (0.10 mg/kg/h iv for 7 days), on top of the optimal treatment, in COVID-19 hospitalized patients. We could screen 131 patients, but due to the predefined strict inclusion and exclusion criteria, only 15 could be randomized to group 1 (n = 7) or group 2 (n = 8). The results of an ad interim safety analysis showed similar overall trends for variables evaluating renal function, coagulation, and inflammation. No adverse events, including hyperkalemia, were found to be associated with nafamostat. Thus, the RACONA study showed a good safety profile of nafamostat, suggesting that it could be usefully used in COVID-19 hospitalized patients.

## 1. Introduction

The COVID-19 pandemic outbreak that emerged in China in 2019 caused by the severe acute respiratory syndrome coronavirus 2 (SARS-CoV-2) has infected more than 769 million people and killed over 6.9 million worldwide (https://www.who.int, accessed on 2 October 2023). On 5 May 2023, after considering the decreasing trend of COVID-19 deaths, the decline in COVID-19 related hospitalizations and intensive care unit admissions, and the high levels of population immunity to SARS-CoV-2, the WHO declared that SARS-CoV-2 no longer constituted a public health emergency of international concern. However, it acknowledged the potential evolution of SARS-CoV-2 and uncertainties on the future spread of the virus and possible outbreaks due to novel mutants (https://www.who.int, accessed on 5 August 2023). Moreover, the vaccination programs did not provide full protection, likely because of waning of antibody titers with time and occurrence of virus variants [1,2,3]. Treatments developed in the last two years improved the course of the disease and prevented severe and fatal complications in most patients, but fragile individuals remain at high risk of respiratory failure, pulmonary embolism, and death [4,5]. Hence, identification of effective treatments remains critically relevant to prevent complications and clinical deterioration in patients with SARS-CoV-2 infection.

Molecular mechanistic research has highlighted that the SARS-Cov-2 virus uses the envelope protein spike (S) to bind to angiotensin-converting-enzyme (ACE)-2, its cellular receptor, which is highly expressed in the lung, particularly in type 2 alveolar epithelial cells and endothelial cells [6]. Upon binding to ACE-2, the virus activates the transmembrane protease serine 2 (TMPRSS2), which allows virus entry into the cell and recruitment of the cell molecular machinery for viral replication and spreading to the surrounding cells [6]. Downstream steps include triggering of the inflammatory pathways and the coagulation cascade, with release of tissue factor and activation of factor VII that favor pulmonary embolism, myocardial damage, and finally disseminated intravascular coagulation [7,8,9].

In 2016, by using a split-protein-based cell–cell fusion assay, Yamamoto et al. showed that the protease inhibitor nafamostat prevented the cell infection by the Middle-East Respiratory Syndrome Coronavirus (MERS-CoV) virus that shares structural similarities with the SARS-CoV-2 [10]. Nafamostat was thereafter discovered to be the most potent known inhibitor of TMPRSS2 [10]. Owing to its action on multiple proteases, nafamostat has been approved for treatment of chronic pancreatitis and disseminated intravascular coagulation in Japan, where it is being widely used in end stage kidney disease patients undergoing chronic dialysis for prevention of filter clotting [11,12,13,14].

In COVID-19 patients, upon binding the tissue factor released by the damaged lung, factor VIIa activates the extrinsic coagulation pathway leading to disseminated intravascular coagulation and pulmonary embolism [9,15]. By inhibiting proteases, including VIIa, at sub-micromolar concentrations, nafamostat could be useful in preventing thrombosis and embolism in COVID-19 patients [16,17]. Furthermore, nafamostat inhibits the epithelial Na^+^ channel (ENaC) and thus Na^+^ reabsorption in the distal nephron and bronchial epithelium. Owing to this latter effect, in 2010 the European Medicines Agency (EMA) granted nafamostat mesylate the status of “orphan drug” for the treatment of children with cystic fibrosis, a genetic disease featuring recurrent pulmonary infections and progressive lung function deterioration due to increased mucus viscosity caused by enhanced eNaC activity. Nonetheless, notwithstanding this approval, nafamostat was never introduced in the EU for patients’ treatment.

Owing to all these properties, we hypothesized that nafamostat could be particularly effective for the treatment of COVID-19 infection. We therefore designed the RAndomized Clinical Trial of NAfamostat (RACONA) study as a prospective randomized, double-blind clinical trial with the specific aim of testing the efficacy and safety of nafamostat in COVID-19 hospitalized patients (clinicaltrial.gov Identifier: NCT04352400, 20 April 2020).

Herein, we report the results of an ad interim safety analysis of RACONA in a cohort of COVID-19 patients who could be recruited between June 2021 and August 2022. The full protocol of the study is provided in the Supplemental Data and only a brief description of the Methods is herein reported.

## 2. Methods

### 2.1. Study Design and Aims

The RACONA is a randomized, double-blind, group-sequential parallel-arm, placebo-controlled trial (allocation ratio 1:1) testing efficacy and safety of nafamostat mesylate on top of best standard of care in hospitalized patients with severe COVID-19 infection. The impact of nafamostat mesylate on several inflammation biomarkers was also assessed as a secondary endpoint.

The trial was registered at clinicaltrial.gov (Identifier: NCT04352400) and was approved by the Ethics Committee of INMI Lazzaro Spallanzani (IRCCS) and the Italian Medicines Agency (AIFA).

### 2.2. Treatments

Treatments (nafamostat mesylate, 0.10 mg/kg/h i.v. dissolved in 5% dextrose, or placebo, i.e., sterile 5% dextrose i.v.) were administered for 7 days as continuous infusion.

To enforce control over bias, an algorithm was specifically created for this study. The algorithm uses a permuted block randomization sequence with stratification (see Supplemental Data for details). Strata were defined by the cross-combination of use of oxygen therapy (nasal duct, mask, etc.) and ongoing treatment with inhibitors of the renin-angiotensin-aldosterone system, as these drugs were suggested to affect outcomes of COVID-19 patients. Investigators and patients were blinded to the treatment administered.

Vital signs, including body temperature, heart rate, respiratory rate, systolic and diastolic blood pressure (BP), oxygen saturation, were monitored daily. Serum K^+^ levels were measured after the first 6 h of infusion and daily during the 7 days of drug administration.

The anonymized data were entered and stored securely in an ad hoc created web-based collection data form (Zucchetti SpA, Lodi, Italy), and stored securely in a server protected with firewalls and passwords.

The primary efficacy outcome was defined as the time-to-clinical improvement, defined as the time from randomization to an improvement of two points (from the status at randomization) on a seven-category ordinal scale. The outcome followed the recommendations of the WHO R&D Blueprint expert group (https://iris.who.int/handle/10665/330680, accessed on 5 August 2023).

### 2.3. Statistical Analysis

#### 2.3.1. Main Analysis

Main analysis was conducted as “as treated”. Results are expressed as mean ± SD, or median and interquartile range, as appropriate. In the case of a skewed distribution, log-transformed data were used. Comparisons were performed with parametric or nonparametric tests (Wilcoxon Mann Whitney), as appropriate; Pearson χ^2^ test was used for analysis of categorical variables. Generalized Linear Models (GLMs) were used to evaluate the relationship between treatments, and time. SPSS for Mac (version 28 for Mac, IBM-SPSS, Bologna, Italy) was used for the statistical analysis and significance was set at *p* < 0.05.

#### 2.3.2. Outcome Evaluation

Bayesian analysis of the response rate was prespecified to estimate the probability of treatment benefit considering the recommendations for trials conducted with a limited sample size in frequentist design [18]. A Beta-binomial model was used to analyze the difference in response rate between arms [18].

The posterior distribution for the difference in proportions outcome requires the estimation of the posterior distribution of the response rate in each arm, separately. It has been computed with the following resampling procedure [19]:

1. A first resampling of the response rate $\pi_{treat}^{*}$ from $\pi_{treat}|X_{treat}$, which is the posterior distribution for the treatment group.
2. A second resampling of $\pi_{control}^{*}$ from $\pi_{control}|X_{2}$.
3. A posterior distribution, for the parameter related to the difference in proportions, has been obtained by calculating $\pi_{treat}^{*}-\pi_{control}^{*}$ from the previously resampled distributions [20].

Resampling procedures were performed using an MCMC estimation algorithm, as indicated in the literature [21], using 3 chains, 5000 iterations, and 1000 adaptations. Computations were performed using OpenBUGS version 3.2 [21] and R version 3.3.2 [22].

#### 2.3.3. Sensitivity Analysis

The inference was expected to be seriously conditioned by the prior choice, because the data points available to estimate the likelihood were only a few. Hence, a sensitivity analysis was performed to assess the robustness of the inferential conclusion with respect to the different prior choices.

Different levels of penalization (discounting) are provided for the historical information using a power prior approach [23] in order to perform a sensitivity analysis on the prior choices.

- Power Prior without discounting (Informative). A $Beta\left(46,6\right)$ for the treatment arm and a $Beta\left(40,10\right)$ for the control arm were derived considering the number of successes reported in the literature in a similar research framework [24].

- Power Prior 50% discounting (Low Informative). The second scenario provides the same informative Beta as previously indicated, also applying a 50% down weight on parameters, to control the effect of prior information on final inference as indicated in the literature [25].
- Power Prior 100% discounting (Uninformative). A $Beta\left(1,1\right)$ prior in both treatments arms has been considered.
  The inference results have been analyzed evaluating the hypothesis that $\pi_{Treat}-\pi_{Control}<0$.

## 3. Results

Patient recruitment started on 4 June 2021, and stopped on 1 August 2022. The results reported herein refer to a safety ad interim analysis performed after recruitment stopped, before opening of the treatment codes, when the investigator was still blinded to randomization. After closing the study on 11 May 2023, we learned that blinded treatments 1 and 2 corresponded to nafamostat and placebo, respectively. 

The baseline demographic and disease characteristics were well balanced in the groups, thus testifying to the effectiveness of the randomization (Table 1).

A total of 131 hospitalized patients with COVID-19 were screened, of whom 62 were found to be eligible for the study (Figure S1). After exclusion of 69 patients because of comorbidities, and/or older age, and/or no need of O_2_ supplementation (see Table S2 for exclusion causes), 15 patients were randomized to group 1 (n = 7) and group 2 (n = 8). One patient withdrew his consent and, therefore, was excluded from the study (Figure S1). No violation or deviation from the protocol was reported.

### 3.1. Safety Outcomes

At the end of treatment (day 7), the clinical and biochemistry parameters showed no differences and no abnormal values (Table 2A). At day 14 of follow-up, higher values of S-creatinine and lower values of eGFR were observed in nafamostat group (*p* = 0.016 and *p* = 0.029, respectively; Table 2B).

**Figure 1:**
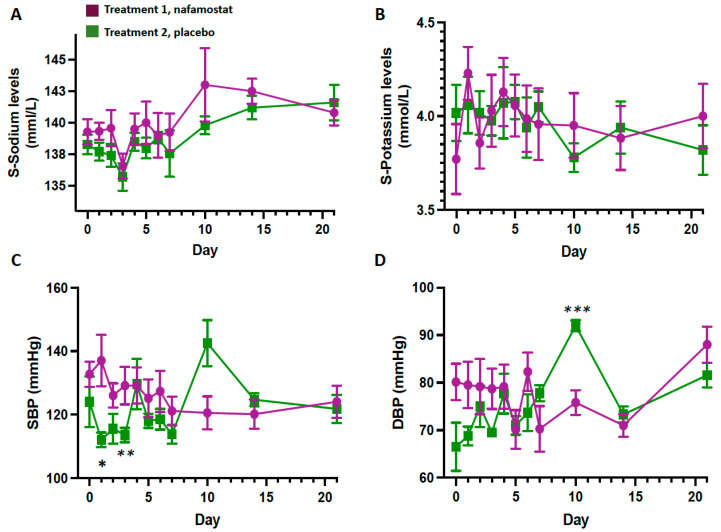
Serum sodium (**A**) and potassium levels (**B**), and systolic (SBP) (**C**) and diastolic blood pressure (DBP) (**D**) in patients receiving treatment 1 (nafamostat) or treatment 2 (placebo). Blinded codes were disclosed after the safety analysis. * *p* = 0.02, ** *p* = 0.01, *** *p* = 0.001, vs. group 1.

At GLM analysis, a decrease of SBP was found in the placebo group at days 1 (−25 mmHg) and 3 (−15 mmHg) (day 1, *p* = 0.02; day 3, *p* = 0.01; Figure 1C), followed by a transient increase of DBP at day 10 (Figure 1D).

**Figure 2:**
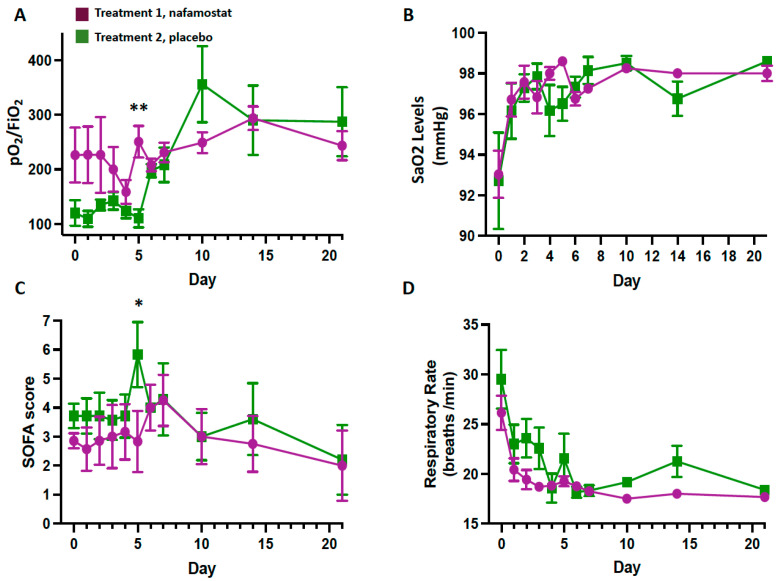
The pO_2_/FiO_2_ (**A**), SaO_2_ levels (**B**), SOFA score (**C**), and respiratory rate (**D**) in patients receiving treatment 1 (nafamostat) or treatment 2 (placebo). Blinded codes were disclosed after the safety analysis. SOFA: Sequential Organ Failure Assessment (including PaO_2_, FiO_2_, mechanical ventilation, platelet counts, Glasgow Coma Scale, Bilirubin, MAP, S-creatinine); * *p* = 0.03; ** *p* = 0.02 vs. group 1.

Trends of pO_2_/FiO_2_ and SaO_2_ were similar, with progressive increase with time (Figure 2A,B). The SOFA score decreased with time in both groups, but a transient increase was seen at day 5 in the placebo group (*p* = 0.032) (Figure 2C). The respiratory rate similarly decreased in both treatment groups (Figure 2D).

**Figure 3:**
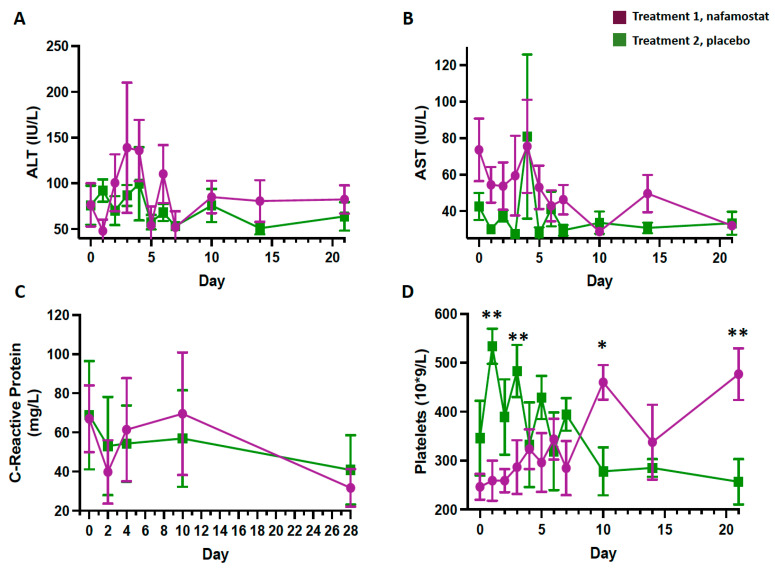
Serum levels of ALT (**A**), AST (**B**), C-reactive protein (**C**), and platelet count (**D**) in patients receiving treatment 1 (nafamostat) or treatment 2 (placebo). Blinded codes were disclosed after the safety analysis. * *p* = 0.01, ** *p* = 0.02, vs. group 1.

The eGFR decreased slightly in the nafamostat group from day 6 to 10 (day 6, *p* = 0.013; days 10 and 14, *p* < 0.001), but returned to normal values. Trends of AST and ALT (Figure 3A,B), CRP (Figure 3C), and S-procalcitonin values were similar between groups. Clinically irrelevant fluctuations were observed in the platelets count in the placebo group (Figure 3D).

### 3.2. Outcomes

Rapid deterioration of lung function leading to death was seen in a 74-year-old female patient, who was initially referred to the Infectious Diseases Unit and then transferred to Pneumology Intensive Unit for dyspnea and interstitial pneumonia, requiring oxygenation via high flow nasal cannulae, remdesivir, azithromycin, ceftriaxone, steroids, and enoxaparin. Nine days after admission cardiac arrest occurred followed by successful cardiopulmonary resuscitation. An increase of D-dimer (1842 µg/L) on day 4, CRP (180 mg/L) on day 5, and troponin I on day 8 (1173 ng/L) was seen, along with apical akinesia with severe depression of LV ejection fraction (EF = 30%) at trans-thoracic echocardiogram. Death occurred on day 10, due to pulseless electrical activity (PEA) unresponsive to CPR and acute kidney failure.

Necropsy findings showed anterior myocardial infarction due to severe narrowing of the left anterior descending coronary artery (LAD), interstitial pneumonia with lymphocytic infiltration, marked injury of pneumocytes with lymphocytic vasculitis and neoangiogenesis, in addition to pronounced damage of the renal tubule-interstitium, adrenal medulla, and liver. Hence, the cause of death was attributed to PEA due to severe hypoxia in the setting of a (previously unknown) tight proximal narrowing of the LAD.

After unblinding, we found that the patient had been allocated to group 2, i.e., the placebo group, but had received treatment for only one day, before the transfer to the ICU.

No drug adverse events were observed during treatment in any patients. In particular, no patient developed cardiovascular disease, including arrhythmia and myocardial infarction, hemorrhages, or hyperkalemia or hyponatremia, defined as S-K^+^ > 5.0 mmol/L and Na^+^ < 130.0 mmol/L, respectively. No differences were found in S-K^+^ or S-Na^+^ average levels between groups (Figure 1A,B).

**Figure 4:**
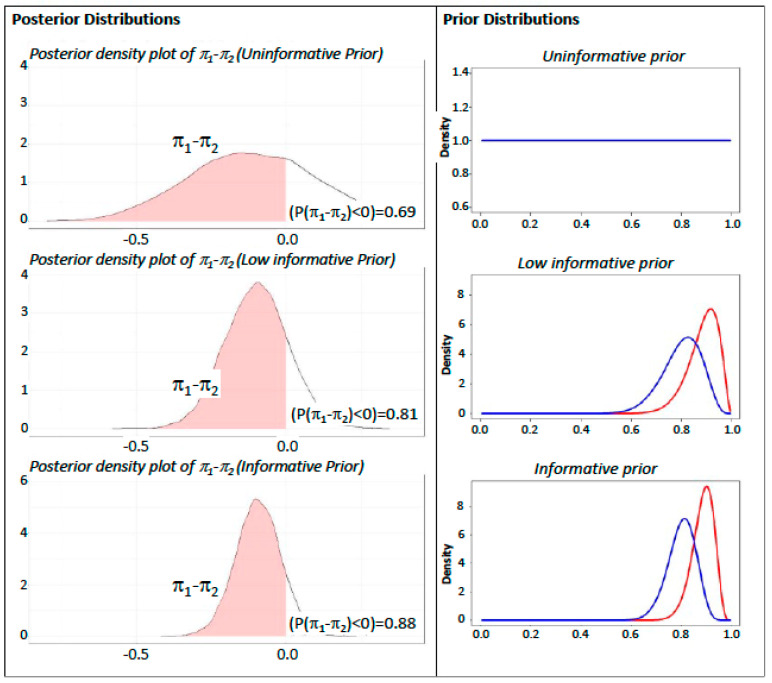
Posterior and prior distributions (blue curve is with penalization for over-optimism and the red curve is without it). The plots refer to the probability that the treatment is effective (i.e., $\;\pi_{control}-\pi_{treat}$ is less than reported) under three scenarios of prior information (Informative, Informative with adjustment for optimism, and Uninformative). Probabilities are $\;\pi_{1}=\;\pi_{control}$ and $\;\pi_{2}=\;\pi_{treat}$.

Fourteen recruited patients completed the follow up for the study outcome (seven in the treatment group and seven in the control group), with the response rates shown in Table 3. The Bayesian evaluation of potential differences in outcome is shown in Figure 4. The Bayesian analysis was conducted to evaluate the probability that the treatment with nafamostat would have a beneficial effect, based on observed data. To evaluate this probability in a Bayesian framework, prior information on the effect of the treatment should be considered in the analysis. Such prior information was incorporated in the analysis by depicting three scenarios, one corresponding roughly to the classical non-Bayesian framework, and two other scenarios where the a priori expectations were mildly or more strongly in favor of the treatment. Prior probabilities were also adjusted in a sensitivity analysis, to correct for a potential over-optimism in the expectations, via down weighting (i.e., penalizing a priori probabilities). Such prior distributions are plotted for clarity on the right side of Figure 4. The distribution functions of the probabilities that the difference in event rate is less than 0 have then been plotted. The estimated curves, which are “blending” prior information with observed data, show a clear signal for a beneficial effect of the nafamostat treatment. In the uninformative scenario the probability that the treatment is effective (difference not zero) is 69%. When prior expectations are taken into account, probabilities that nafamostat treatment is effective are stronger (in low informative prior scenario probability of an effective treatment is 88%, and in the informative prior scenario with down weight is 81%).

## 4. Discussion

The RACONA study was conceived in 2020, soon after the breakout of COVID-19 in China and its fast spreading that caused more than 15 thousand deaths, between 23 February and 30 April, in Italy (https://www.salute.gov.it/, accessed on 10 August 2023). Starting from the identification of the role of TMPRSS2 in virus spread and recruitment of the cell machinery, and of the effectiveness of camostat, a TMPRSS2 inhibitor in blunting SARS-CoV-2 entry into cells [6], our attention was caught by nafamostat, a protease inhibitor 10-thousand-fold more potent than camostat on TMPRSS2, which was known to be well-tolerated in end stage kidney disease patients on chronic hemodialysis in Japan (Y.K., personal communication). This was also because, in contrast to camostat, which has anti-fibrinolytic effects and can cause eosinophilic pneumonia, nafamostat has anticoagulant properties that seemed attractive for preventing the thrombotic events associated with COVID-19 infection [26].

Based on these premises, we conceived the RACONA study to evaluate the efficacy and safety of nafamostat in COVID-19 infected patients. However, owing to several hurdles, including the fact that nafamostat had never entered the EU for clinical use before, approval of the study protocol took several months, thus allowing us to start recruitment only in May 2021. In the meantime, other treatments, including antiviral drugs, monoclonal antibodies, and vaccines, had been approved by the Italian regulatory agency AIFA. Notwithstanding the active involvement of three units, i.e., Pneumology, Infectious Diseases, and Internal Medicine, at our University Hospital, this rendered enrollment extremely difficult, and explains why we could enroll only 15 patients, a cohort that, according to our prior calculations, was underpowered to detect differences of treatment efficacy between groups. Considering, however, that nafamostat was never used before in patients outside of Japan and South Korea, we thought it important to perform a safety analysis of this drug. The latter showed that there were no major adverse events, including hemorrhages, or cardiovascular disease, including arrhythmia and myocardial infarction.

When designing the study, we paid attention to monitoring serum ion levels daily, because nafamostat inhibits the epithelial Na^+^ channel (eNaC) and, therefore, could theoretically cause hyperkalemia and hyponatremia [27,28,29]. It is worth noting that none of our patients developed any clinically relevant changes in an extended panel of biochemical markers, including serum K^+^ and Na^+^ levels (Figure 1A,B). This piece of information can be useful for patients who are receiving drugs, such as the mineralocorticoid receptor antagonists that act via eNaC, and, therefore, could have hyperkaliemia.

In a GLM analysis that included time in addition to treatment, we observed similar overall trends for most examined variables, with only transiently clinically differences between groups. As mentioned above, a patient of group 2 developed rapid deterioration of the lung function, which required transfer to ICU, with following death. An independent adverse event adjudication committee judged this death to be unrelated to treatment, but due to COVID-19 complications that were commonly seen at that time. After stopping the study and unblinding the treatment code, the patient was found to be allocated to the placebo group. On the whole, the data obtained support the concept that nafamostat is safe when administered in patients with no contraindications as severe CKD or low platelet count.

Interestingly, after we registered the RACONA study protocol in clinicaltrials.gov in April 2020, the interest for nafamostat rapidly grew in many countries, leading to a number of trials with a similar design in hospitalized patients for COVID-19 pneumonia.

A study with the same aims and methodology was posted in www.clinicaltrials.gov (accessed on 10 August 2023) by a Korean group (June 2020; Identifier: NCT04418128), but to date it is reported as “not yet recruiting”. Six other trials have been completed, and four are still ongoing (Table 4 and Table 5). In a randomized open-label trial performed in Russia in high-risk COVID-19 patients (Identifier: NCT04623021), the time to clinical improvement was reported to be shorter with nafamostat than with placebo. Phlebitis due to continuous i.v. infusion was the only side effect observed in the nafamostat group [24].

In an open label, controlled Scottish trial (DEFINE trial, NCT04473053), nafamostat was associated with more hyperkalemia (in 14.3% of patients) and deterioration of renal function [31], two adverse effects that we did not observe in RACONA.

No results are available from the two other open-label, controlled studies reported to be completed in Korea (NCT046288143 clinicaltrials.gov) and India (https://trialsearch.who.int (accessed on 7 August 2023), CTRI/2020/06/026220).

In a retrospective cohort study performed in Japan in patients hospitalized for COVID-19 and administered with nafamostat mesylate within 2 days of admission, no differences were found in-hospital mortality between nafamostat and optimal treatment for COVID-19; no information on safety was provided [35].

Considering that use of nafamostat in COVID-19 patients has been proposed starting from solid mechanistic premises, and that the RACONA study has a strong randomized, double-blind, placebo-controlled study design, we believe that sharing our data with the scientific community is important at a time when nafamostat treatment outside of Japan and South Korea has been scarce.

The present results showed no serious adverse events with nafamostat, indicating that, when infused i.v. for 7 days, it is safe in COVID-19 patients. Moreover, results of our Bayesian analysis of efficacy evidenced a signal for a beneficial effect of the drug under different assumptions (Figure 4). Thus, these results indicate that nafamostat on top of current treatments for COVID-19 could be useful to enrich the therapeutic armamentarium against Sars-CoV-2, a disease that still affects a number of fragile people, with requirement of hospitalization and high-level health care.

### Limitations of the Study and Perspectives

The major limitation of the study is the small sample size that was due to the difficulties in the enrollment and the large use of other competing treatment trials, i.e., antiviral drugs, monoclonal antibodies, and vaccines, which were meanwhile approved by AIFA for cure of the COVID-19 patients. Hence, since the RACONA study was underpowered to detect differences of treatment efficacy between groups, only a safety analysis was performed. However, following a Bayesian approach, we were able to detect a signal for a beneficial effect of the drug under different assumptions. Thus, to investigate the efficacy in more severely ill patients with COVID-19 infection, such as mechanically ventilated patients, further studies are necessary and worth pursuing.

## 5. Conclusions

Identification of new drugs, as well as repurposing of approved drugs that hold information on safety and tolerability accumulated over 30 years, are needed to contrast the changes of pandemics that follow the development of new variants and the waning of immunization. In the RACONA study, nafamostat showed a good safety profile and, therefore, could represent an effective tool, particularly against those variants that are more dependent on TMPRS2 (e.g., omicron vs. delta variant).

## Figures and Tables

**Table 1:** Demographic and disease characteristics at baseline.

Variable	Group 1 Nafamostat (n = 7)	Group 2 Placebo (n = 7)	All Patients (n = 14)
Male sex, n (%)	6 (85%)	4 (57%)	10 (71)
Age, years	64 [46–68]	58 [58–77]	62 [57–74]
Body temperature, °C	37.5 (36.2–38.5)	37.7 (36.9–39.0)	37.5 [36.8–38.7]
SBP, mmHg	133 [130–140]	139 [118–154]	135 [127–143]
DBP, mmHg	80 [70–92]	84 [63–91]	82 [69–91]
Heart rate/min	88 [72–95]	96 [81–103]	90 [80–102]
Respiratory rate/min	28 [23–30]	29 [23–33]	28 [23–30]
Seven-category ordinal scale, score	4 [4–5]	5 [5–5]	5 [4–5]
SOFA score, Units	3 [2–3]	4 [3–5]	3 [2–4]
C-reactive protein, mg/L	59 [29–118]	40 [12–150]	49 [13–121]
D-dimer, μg/L	189 [150–259]	286 [156–657]	218 [154–513]
S-Creatinine, μmol/L	70 [64–87]	62 [53–104]	68 [54–87]
eGFR, mL/min/1.73 m^2^	92 [80–106]	98 [68–104]	94 [80–103]
Fibrinogen, g/L	4.90 [4.50–6.80]	4.30 [3.60–4.80]	4.65 [4.27–5.20]
PT, s	98 [96–101]	98 [87–108]	98 [87–106]
INR	1.07 [1.04–1.11]	1.08 [1.04–1.14]	1.08 [1.04–1.12]
Platelets, 10^9^/L	237 [202–304]	268 [132–355]	251 [185–316]
SatO_2_, %	93 [90–94]	93 [85–99]	93 [90–97]
pO_2_/FiO_2_ ratio	161 [110–379]	98 [73–162]	121 [97–231]
Procalcitonin, µg/L	0.08 [0.05–0.14]	0.25 [0.04–0.93]	0.08 [0.04–0.65]
PSI, Units	84 [56–96]	98 [93–107]	94 [75–100]
CURB-65, Units	1 [0–2]	2 [1–2]	1 [1–2]
S-Ferritin, µg/L	1884 [554–4635]	1358 [943–1721]	1358 [886–2507]
S-K^+^, mmol/L	4.0 [3.4–4.1]	4.3 [3.9–4.4]	4.0 [3.7–4.3]
S-Na^+^, mmol/L	138 [137–141]	139 [137–140]	138 [137–141]

Median (IQR). CURB-65: Confusion, Uremia, Respiratory rate, BP, age ≥ 65 years); PSI: Pneumonia Severity Index; SOFA: Sequential Organ Failure Assessment.

**Table 2:** Outcomes at (A) day 7 and (B) day 14.

Variable	Group 1 Nafamostat (n = 7)	Group 2 Placebo (n = 7)	*p*
**(A) Outcomes at day 7**			
Body temperature, °C	36.2 [36.0–38.5]	36.1 [36.0–36.8]	0.527
SBP, mmHg	115 [104–120]	130 [109–150]	1.000
DBP, mmHg	70 [66–72]	80 [65–89]	0.476
Heart rate, bpm	79 [58–95]	71 [60–81]	0.476
Respiratory rate/min	18 [18–18]	18 [16–20]	1.000
Seven-category ordinal scale, score	3 [1–6]	6 [4–6]	0.295
SOFA score, Units	4 [1–7]	5 [1–7]	0.927
C-reactive protein, mg/L	33 [12–144]	48 [2.9–120]	0.876
D-dimer, µg/L	163 [152–248]	626 [300–4279]	0.012
S-Creatinine, μmol/L	106 [92–136]	66 [49–94]	0.109
eGFR ml/min/1.73 m^2^	59 [45–89]	95 [63–109]	0.257
S-Fibrinogen, g/L	6.00 [4.00–7.25]	3.70 [2.80–6.10]	0.164
PT, s	93 [79–104]	89 [76–103]	0.648
INR	1.11 [1.04–1.18]	1.10 [1.06–1.20]	1.000
Platelets, 10^9^/L	495 [308–625]	259 [228–270]	0.048
SatO_2_, %	97 [97–98]	98 [97–100]	0.230
pO_2_/FiO_2_	233 [165–294]	172 [156–257]	0.648
S-Procalcitonin, µg/L	0.08 [0.04–0.33]	0.06 [0.04–0.21]	1.000
PSI, Units	83 [50–113]	97 [58–114]	0.648
CURB-65, Units	2 [1–2]	1 [1–3]	0.527
S-Ferritin, µg/L	1003 [287–2409]	894 [607–1319]	1.000
S-K^+^, mmol/L	4.0 [3.6–4.2]	4.0 [3.7–4.4]	1.000
S-Na^+^, mmol/L	139 [135–143]	138 [136–139]	0.648
**(B) Outcomes at day 14**			
Body temperature, °C	36.1 [36.0–36.7]	36.0 [36.0–37.1]	0.610
SBP, mmHg	115 [102–123]	135 [121–171]	1.000
DBP, mmHg	78 [71–83]	92 [87–95]	0.476
Heart rate, bpm	80 [67–80]	83 [66–111]	0.476
Respiratory rate/min	18 [16–19]	18 [18–27]	0.171
Seven-category ordinal scale, score	1 [1–4]	4 [1–6]	0.432
SOFA score, Units	1 [1–6]]	2 [1–7]	1.000
C-reactive protein, mg/L	10 [3–10]	10 [10–108]	1.000
D-dimer, µg/L	274 [161–453]	421 [192–777]	0.063
S-Creatinine, μmol/L	92 [89–92]	65 [49–70]	0.016
eGFR ml/min/1.73 m^2^	83 [76–89]	102 [93–105]	0.029
S-Fibrinogen, g/L	5.1 [3.6–7.7]	4.7 [3.1–5.9]	0.413
PT, sec	87 [67–101]	104 [90–107]	0.556
INR	1.12 [1.02–1.23]	1.04 [1.03–1.12]	1.000
Platelets, 10^9^/L	425 [267–490]	206 [166–425]	0.413
SatO_2_, %	98 [97–98]	98 [93–98]	0.762
pO_2_/FiO_2_	314 [208–357]	219 [108–542]	0.476
S-Procalcitonin, µg/L	0.04 [0.04–0.11]	0.04 [0.04–2.30]	1.000
PSI, Units	73 [52–82]	108 [63–160]	0.762
CURB-65, Units	1 [1–2]	2 [1–2]	0.730
S-Ferritin, µg/L	736 [62–736]	404 [281–404]	1.000
S-K^+^, mmol/L	3.9 [3.8–4.3]	3.7 [3.6–4.0]	0.905
S-Na^+^, mmol/L	142 [140–145]	143 [139–143]	1.000

Mean ± SD, or median (range) as appropriate. Values of C-reactive protein, S-Ferritin, S-Procalcitonin, and CURB-65 refer to day 28.

**Table 3:** Number and percentages of responses in treatment and control arm. The 95% Credible Intervals are reported for the posterior distribution $\;\pi_{Treat}-\pi_{Control}$ and for predictive posterior estimates provided in Informative, Low informative, and Uninformative scenarios.

	Control (n = 7)	Treatment (n = 7)	
	29% (2)	43% (3)	
	Predictive Posterior Estimates (95% CI)		$\pi_{control}-\pi_{treat}\;(95\%\;\mathrm{CI})$
Informative	5 (3; 7)	6 (3; 7)	−0.09 (−0.24; 0.05)
Low Informative	5 (2; 7)	6 (3; 7)	−0.1 (−0.31; 0.11)
Uninformative	2 (0; 6)	3 (0; 6)	−0.11 (−0.52; 0.32)

**Table 4:** Completed trials of nafamostat mesylate for treatment of COVID-19 patients.

Authors, Journal	ID, Registration Date, Acronym (If Available)	Country	Trial Design	Primary Endpoint	Patients (n)	Dose	Outcome/Side Effects
Rossi G.P. et al. *Submitted* [30]	NCT04352400 Posted April 2020 RACONA	Italy	Prospective randomized, double-blind, placebo-controlled	Clinical efficacy and safety	Target number: 256 Enrolled: 15	0.10 mg/kg/h i.v. for 7 days	No adverse events, including no hyperkalemia
Quinn T.M. et al. eBioMedicine 2022 [31]	NCT04473053 Posted 16 July 2020 DEFINE	Scotland	Prospective, open label, controlled, multicenter	Safety and tolerability	In-hospital patients (21 vs. 21 SoC)	0.2 mg/Kg/h i.v. for 7 days	No serious adverse events. Hyperkalemia (14.3% of patients) and higher plasma creatinine levels (mean difference 10.57 umol/L, 95% CI 2.43–18.92).
Unpublished No available results [32]	CTRI/2020/06/026220 Posted July 2020	India	Prospective, randomized open-label, controlled	Proportion of patients with clinical improvement at day 14	In-hospital patients	0.1 mg/kg/h i.v. for 10 days	N.A.
Zhuravel S.V. et al. *EClinical Medicine* 2021 [24]	NCT04623021 Posted 10 November 2020	Russia	Prospective, randomized open-label (nafamostat vs. SOC), multicenter	Time to clinical improvement	In-hospital patients * (53 vs. 51 vs. SOC)	0.2 mg/kg/h for 10 days or discharge	No overall difference in primary endpoint. In high-risk patients (NEWS > 7) nafamostat was superior to SOC (11 vs. 14 days; RR, 2.89; 95% CI, 1.17 to 7.14; *p* = 0.012). No serious adverse events. Phlebitis (13% vs. 3%)
Unpublished No available results [33]	NCT04628143 Posted 13 November 2020	South Korea	Prospective, open-label, controlled	Time to clinical improvement	In-hospital patients vs. SOC (total 31)	N.A.	N.A.
Soma T. et al. *Jpn J Infect Dis* 2022 [34]	N.A.	Japan	Retrospective single centre	Clinical deterioration	Moderate-risk patients ** (31 vs. 33 conservative supportive treatment)	0.06–0.2 mg/Kg/h i.v. for 5 days	No differences in deterioration or death Hyperkalemia, i.e., >5 mmol/L, (21% vs. unknown) No need additional treatment after discontinuation
Inokuchi R. et al. *J Clin Med* 2022 [35]	N.A.	Japan	Retrospective observational	Mortality	In-hospital patients (121 vs. 15859)	N.A.	No difference in mortality. No side effects reported.

SOC: standard of cure; * Requiring nasal high-flow oxygen therapy and/or non-invasive mechanical ventilation; ** Evidence of acute lower respiratory disease on clinical assessment or imaging, and SpO_2_ ≥ 94% on room air. Severe was defined as SpO_2_ < 94% on room air, PaO_2_/FiO_2_ < 300 mmHg, respiratory rate > 30 breaths/min, or lung infiltrates > 50%.

**Table 5:** Ongoing trials of nafamostat mesylate for treatment of COVID-19 patients.

ID Registration	Country	Trial Design	Primary Outcome Measure	Patients (Condition, Dose, Target Number and Control)
NCT05555641 27 September 2022	Wuhan, Hubei, China,	Randomized Parallel Assignment	Incidence of severe bleeding	ECMO anticoagulated critically ill patients Dose and target number N.A. Unfractionated heparin
NCT04390594 15 May 2020	Senegal	Randomized Parallel Assignment	SARS-CoV-2 viral load level at day 7	In-hospital patients Dose: 0.1–0.2 mg/kg/h Target number: total 186 SOC
NCT04483960 23 July 2020 ASCOT ADAPT	Melbourne	Randomized Factorial assignment Open label	Death from any cause or requirement of new intensive respiratory support or vasopressor/inotropic support	In-hospital patients Dose: 0.2mg/kg/hour Target number: N.A. Enoxaparin, dalteparin or tinzaparin
NCT04871646 4 May 2021	Republic of Korea	Double-blind Randomized Parallel Assignment Multicenter	Time to recovery	In-hospital patients Dose: N.A. Target number: N.A. SOC

SOC: standard of cure.

## Data Availability

Data available on request due to restrictions on privacy.

## Supplementary Materials

The following supporting information can be downloaded at: https://www.mdpi.com/article/10.3390/jcm12206618/s1, Materials contain the Protocol of RACONA Study [6,11,30,36,37,38,39,40,41,42,43,44,45,46,47]; Table S1: Seven-category ordinal scale; Table S2. Inclusion and exclusion criteria; Table S3. Flow chart of the study; Table S4. Laboratory tests; Table S5. Secondary Outcomes; Table S6. Secondary biomarker endpoints; Table S7. Group-sequential design; Figure S1. Flow chart of the RACONA Study; Annex S1: CONSORT chart.
