# Supplementary material for: RAndomized Clinical Trial Of NAfamostat Mesylate, A Potent Transmembrane Protease Serine 2 (TMPRSS2) Inhibitor, in Patients with COVID-19 Pneumonia
Source: J Clin Med. 2023 Oct 19;12(20):6618. doi: 10.3390/jcm12206618 (PMC10607860; doi:10.3390/jcm12206618)
Supplement: Supplementary file 1 [file jcm-12-06618-s001.zip › jcm-2590613-supplementary.pdf]

RAndomized Clinical Trial Of NAfamostat Mesylate,
A Potent Transmembrane Protease Serine 2 (TMPRSS2) Inhibitor,
in patients with Covid-19 pneumonia: Safety
SUPPLEMENTAL DATA

Teresa Maria Seccia[1], Tungalagtamir Shagjaa [1], Margherita Morpurgo[2], Brasilina Caroccia[1], Viola Sanga[1], Sonia Faoro[3], Francesca Venturini[3], Girolama Iadicicco[3], Sara Lococo[4], Maria Mazzitelli[5], Filippo Farnia[6], Paola Fioretto[6], Yusuke Kobayashi[7], Dario Gregori[8], Gian Paolo Rossi[1]

[1]Internal Emergency Medicine Unit, Department of Medicine-DIMED University of Padua, Specialized Center for Blood Pressure Disorders-Regione Veneto, Padua;
[2]Department of Pharmaceutical and Pharmacological Sciences, University of Padua;
[3]Pharmacy, [4]Pneumology, [5]Infectious Diseases, [6]Internal Medicine 3, University Hospital of Padua; [7]Yokohama City University, Japan;
[8]Biostatistics, Epidemiology and Public Health Unit, University of Padua, Italy

Running title: Nafamostat in Covid-19 patients

Conflict-of-interest statement: The authors declare no conflict of interest

Corresponding author:
Prof. Gian Paolo Rossi, MD, FACC, FAHA.
Internal Emergency Medicine Unit, Department of Medicine-DIMED
University of Padua,
& Specialized Center for Blood Pressure Disorders-Regione Veneto,
University Hospital
Via Giustiniani, 2
35126 Padova, Italy
Phone: +39-049-821-2279 or 7821
E-mail: gianpaolo.rossi@unipd.it

Protocol of RACONA Study

Study design and aims

The RACONA is a randomized, double-blind, placebo-controlled on top of best standard of care, phase 2 trial, with group-sequential parallel-arms. The primary aim was to assess the clinical efficacy of nafamostat mesylate in hospitalized patients with Covid-19 infection, with the efficacy defined as at least a 2-point improvement in a clinically validated seven-category ordinal scale (categories ranging from 1 to 7, with higher categories indicating a worse condition; Table S1). The secondary objective was to evaluate the effects of the drug on the clinical safety and the impact on inflammation biomarkers. The trial, registered at clinicaltrial.gov in April 2020 (Identifier: NCT04352400), received approval from Ethic Committee of INMI Lazzaro Spallanzani (IRCCS) and Italian Medicines Agency (AIFA).

Patients

Patients with age 18-85 years who were hospitalized because of Covid-19 infection, confirmed by a positive polymerase-chain-reaction (PCR) test and imaging, were eligible for enrollment. Inclusion and exclusion criteria are reported in Table S2. Briefly, inclusion criteria entail written signed informed consent form; body temperature >37.3℃, or history of fever in the two weeks before hospital admission, or use of fever-killing medications, and abnormal oxygenation (any of the following: *i)* oxygen saturation ≤95% on room air; *ii)* PaO2/FiO2 ratio ≤300 mmHg; *iii)* need of oxygen supplementation; respiratory rate (RR) ≥18 beats/min. Patients were excluded if *i)* pregnant or lactating females; *ii)* unwilling or not able to perform the study; *iii)* eGFR < 30 ml/min/m2 (CKD EPI formula); *iv)* liver disease (Child Pugh score ≥ 10); *v)* participation to a clinical trial with an investigational product; v*i)* history of anaphylactic shock; v*ii)* unstable hemodynamics; *viii)* active bleeding; *x)* malignancy; *xi)* chronic interstitial infiltration that can mislead diagnosis of covid-19 disease at imaging; *xii)* hyperkalemia.

Treatments

The study comprised 2 treatment groups: active group (nafamostat mesylate, 0.10 mg/kg/h i.v. dissolved in 5% dextrose); control group (placebo, sterile 5% dextrose i.v.). Nafamostat was kindly provided by Kyoso Mirai Pharma, Japan.

Nafamostat, an ester conjugate of p-guanidinobenzoic acid (GBA) and 6-amidino-2-naphthol, inhibits the activity of TMPRSS2 with an IC50 of between 5 and 55 nM [1,2]. The half-maximal inhibitory concentration (IC50) of nafamostat in preventing infection of alveolar epithelial cells by SARS-CoV-2 is in the range of 5-10 nM.6 The steady-state plasma concentrations of nafamostat when infused to patients with DIC at 0.1 mg/kg/h or 0.2 mg/kg/h is reported to be between 14 and 130 ng/mL, which exceeds the IC50 for TMPRSS2 inhibition.

With a half-life of 8 minutes[3] nafamostat mesylate is rapidly eliminated from the blood [4], being metabolized into inactive compounds by arylesterases and carboxylesterase 2 in the blood and liver [5].

Hence, nafamostat administration was planned as a continuous i.v. infusion over 24 hours for seven days at a dose of 0.10 mg/Kg/h. Decrease (down to 0.05 mg/Kg/h) or increase (up to 0.20 mg/kg) of the dose was allowed, depending on adverse effects, e.g. mild to moderate hyperkalemia, or persistence of fever or dyspnea. Detectable levels of the metabolite support a rapid breakdown in vivo.

Eligible patients, as defined above, were randomly allocated (1:1) to the active or control groups at baseline (day 1). To enforce control over bias, an algorithm was specifically created for this study. The algorithm uses a permuted block randomization sequence with stratification [6] Strata were defined by the cross-combination of use of oxygen therapy (nasal duct, mask, etc.) and ongoing treatment with inhibitors of the renin-angiotensin-aldosterone system, as these drugs were suggested to affect outcomes of covid-19 patients.[7–9] Investigators and patients were blinded to the treatment administered.

After screening (day -1), the patients were treated with nafamostat mesylate or placebo starting from day 1 until day 7 as specified below. Follow-up visits were done at a daily basis for the first seven days, and then on day 10, 14 and 28 to asses safety (Table S3). Vital signs, including body temperature, heart rate, respiratory rate, systolic and diastolic blood pressure (BP), oxygen saturation, were reported daily. Clinical examination included assessment of SOFA, PSI and CURB65 score and registration of adverse events, if any. Chest X-ray or CT were performed at the screening, and days 7 and 28 after the treatment (Table S3).

Blood pressure was monitored noninvasively with automated devices throughout the infusion. Since hyperkalemia was occasionally found after nafamostat treatment, serum K+ levels were measured after the first 6 hours of infusion and daily during the 7 days of drug administration (Table S4).

Data collection

The anonymized data were entered and stored securely in an ad hoc created web-based collection data form (Zucchetti SpA, Lodi, I), and stored securely in a server protected with firewalls and passwords.

Concomitant treatments

Patients were allowed to continue their treatment for pre-existing conditions, including ACE-inhibitors, angiotensin II AT1 receptor blockers (ARB). Mineralocorticoid receptor antagonists (MRA), which act via eNaC, were also allowed, but utmost attention was given to detect the onset of hyperkalemia that, if occurring, would lead to their immediate withdrawal and use of polystyrene sulfonate. Antiplatelet drugs, if needed, and ongoing treatments with antiviral and anti-inflammatory drugs, if ongoing, were allowed.

Outcome measures

The primary efficacy outcome was defined as the time-to-clinical improvement, defined as the time from randomization to an improvement of two points (from the status at randomization) on a seven category ordinal scale. The outcome followed the recommendations of the WHO R&D Blueprint expert group [10].

The secondary efficacy outcomes included 1) the rate of responders, defined as patient showing improvement of at least two points in seven-category ordinal scale at day 7, 10 ,14, 21 and 28; 2) the proportion of patients who progressed to critical illness/death; 3) change in pO2/FiO2 ratio over time; 4) change Sequential Organ Failure Assessment score (SOFA score) over time; 5) improvement at imaging, as assessed at chest CT, 6) duration of hospitalization (days) in survivors, 7) proportion of patients who require mechanical ventilation and duration of mechanical ventilation (Table S5).

The safety outcomes entailed adverse events (fatal and non-fatal), premature discontinuation of treatment due to adverse events, proportion of patients who develop arrhythmia, or myocardial infarction, or other cardiovascular disease not present at the baseline, hyperkalemia defined as S-K+ > 5.0 mmol/L, or hyponatremia defined as Na+< 130.0 mmol/L, hemorrhages (Table 1).

The safety biochemical outcomes included evaluation of changes over time of all investigated parameters (renal and liver function, blood cells, coagulation, and inflammation)(Tables S5 and S6).

Setting. University Hospital of Padova.

Sample size. The study was designed as an event-driven group-sequential parallel-arm trials [11], with two interim analyses, with non-binding formal control for futility. The primary objective of the study was to detect a statistically significant difference in time to 2-point improvement of the seven-category ordinal scale for the nafamostat arm relative to the control arm, using the classical Lachin design [12]. As the divergence between cases following a non-fatal vs a fatal clinical course started to occur on average after 7-10 days, it was anticipated that the median time for starting detecting a difference between the nafamostat group and the placebo group fall in between 7 to 10 days, with a hazard ratio between the treatment arms of 0.70 [13] We estimated that 200 events are needed at the final analysis, assuming a censoring time at 30 days, an overall study duration of 6 months and a uniform overall accrual time of 4 months, with a symmetric two-sided alpha level of 0.05 and a power

of 0.80. This number of events corresponds to an expected total sample size of 256 patients. Futility analyses performed at the time of interim analysis (Table S7). Computations have been performed using the R System [14, 15]and the gsDesign libraries [13].

References

1 Hoffmann M, Kleine-Weber H, Schroeder S, Krüger N, Herrler T, Erichsen S, *et al.* SARS-CoV-2 Cell Entry Depends on ACE2 and TMPRSS2 and Is Blocked by a Clinically Proven Protease Inhibitor. *Cell* 2020; 181:271-280.e8.

2 Hoffmann M, Schroeder S, Kleine-Weber H, Müller MA, Drosten C, Pöhlmann S. Nafamostat Mesylate Blocks Activation of SARS-CoV-2: New Treatment Option for COVID-19. *Antimicrob Agents Chemother* 2020; 64. doi:10.1128/AAC.00754-20

3 Maruyama Y, Yoshida H, Uchino S, Yokoyama K, Yamamoto H, Takinami M, *et al.* Nafamostat mesilate as an anticoagulant during continuous veno-venous hemodialysis: A three-year retrospective cohort study. *International Journal of Artificial Organs* 2011; 34:571–576.

4 Cao Y, Zhang M, Yu D, Shao J, Chen Y, Liu X. A method for quantifying the unstable and highly polar drug nafamostat mesilate in human plasma with optimized solid-phase extraction and ESI-MS detection: more accurate evaluation for pharmacokinetic study. *Anal Bioanal Chem* 2008; 391:1063–71.

5 Yamaori S, Fujiyama N, Kushihara M, Funahashi T, Kimura T, Yamamoto I, *et al.* Involvement of human blood arylesterases and liver microsomal carboxylesterases in nafamostat hydrolysis. *Drug Metab Pharmacokinet* 2006; 21:147–55.

6 Broglio K. Randomization in clinical trials: Permuted blocks and stratification. *JAMA - Journal of the American Medical Association* 2018; 319:2223–2224.

7 Vaduganathan M, Vardeny O, Michel T, Mcmurray J V., Pfeffer MA, Solomon SD. Renin – Angiotensin – Aldosterone System Inhibitors in Patients with Covid-19. *New Engl J Med* 2020; :1-7 DOI: 10.1056/NEJMsr2005760.

8 Rossi GP, Sanga V, Barton M. Potential harmful effects of discontinuing ACE-inhibitors and ARBs in COVID-19 patients. *Elife* 2020; 9_e57278:1–8.

9 Zhang P, Zhu L, Cai J, Lei F, Qin J, Xie J, *et al.* Association of Inpatient Use of Angiotensin Converting Enzyme Inhibitors and Angiotensin II Receptor Blockers with Mortality Among Patients with Hypertension Hospitalized With COVID-19. *Circ Res* 2020; CIRCRESAHA.120.317134.

10 World Health Organization. WHO R&D Blueprint Informal consultation on prioritization of candidate therapeutic agents for use in novel coronavirus 2019 infection. Geneva: ; 2020.

11 Lesaffre E. "The Randomized Controlled Trial: Methodological Perspectives." In: *Understanding Evidence-Based Rheumatology.* ; 2014. pp. 159-178.

12 Lachin JM, Foulkes. MA. Evaluation of sample size and power for analyses of survival with allowance for nonuniform patient entry, losses to follow-up, noncompliance, and stratification. *Biometrics* 1986; 42:507–519.

13 Zhou F, Yu T, Du R, Fan G, Liu Y, Liu Z, *et al.* Clinical course and risk factors for mortality of adult inpatients with COVID-19 in Wuhan, China: a retrospective cohort study. *The Lancet* 2020; 6736:1–9.

14 R_Core Team. R: A language and environment for statistical computing. R Foundation for Statistical Computing. 2020.

15 Keaven Anderson K. gsDesign: Group Sequential Design. *30-1, package version Https://CRANR-project.org/package=gsDesign* 2016.

Participants

Internal Emergency Medicine Unit, Department of Medicine-DIMED University of Padua, Specialized Center for Blood Pressure Disorders-Regione Veneto, Padua:
Gian Paolo Rossi
Teresa Maria Seccia
Tungalagtamir Shagjaa
Brasilina Caroccia
Viola Sanga
Department of Pharmaceutical and Pharmacological Sciences, University of Padua
Margherita Morpurgo
Infectious Diseases, University Hospital of Padua:
Annamaria Cattelan
Maria Mazzitelli
Internal Medicine 3, University Hospital of Padua:
Paola Fioretto
Roberto Vettor
Filippo Farnia
Pneumology, University Hospital of Padua:
Andrea Vianello
Sara Lococo
Beatrice Molena
Unit of Pharmacy, University Hospital of Padua
Francesca Venturini
Sonia Faoro
Girolama Iadicicco
Biostatistics, Epidemiology and Public Health Unit, University of Padua, Italy
Danila Azzolina
Dario Gregori
Yokohama City University, Japan
Yusuke Kobayashi
Zucchetti SpA, Lodi, Italy
Marco Piccolo

Table S1. Seven-category ordinal scale

Score	Descriptor
1	Not hospitalized with resumption of normal activities
2	Not hospitalized, but unable to resume normal activities
3	Hospitalized, not requiring supplemental oxygen
4	Hospitalized, requiring supplemental oxygen
5	Hospitalized, requiring nasal high-flow oxygen therapy, noninvasive mechanical [illegible]
6	Hospitalized, requiring ECMO, invasive mechanical ventilation, or both
7	Death

Table S2. Inclusion and exclusion criteria

**Inclusion criteria**
Written signed informed consent form;<br>Body temperature >37.3℃, or history of fever in the two weeks before hospital admission, or use of fever-killing medications,<br>and<br>abnormal oxygenation (any of the following: i) oxygen saturation ≤95% on room air; ii) PaO2/FiO2 ratio ≤300 mmHg; iii) need of oxygen supplementation;<br>Respiratory rate (RR) ≥18 beats/min
**Exclusion criteria**
Pregnant or lactating females;<br>Unwillingness or not ability to perform the study;<br>eGFR < 30 ml/min/m2 (CKD EPI formula);<br>Liver disease (Child Pugh score ≥ 10);<br>Participation to a clinical trial with an investigational product;<br>History of anaphylactic shock;<br>Unstable hemodynamics;<br>Active bleeding;<br>Malignancy;<br>Chronic interstitial infiltration that can mislead diagnosis of covid-19 disease at imaging;<br>Hyperkalemia.

Table S3. Flow-chart of the study

Task	Enrolment	Randomization	Treatment										
	Days												
	-1	0	1	2	3	4	5	6	7	10	14	21	28
Informed consent	X												
Inclusion/exclusion criteria	X												
Demographics[a]	X												
Clinical history	X												
Randomization		X											
Seven-category ordinal scale	X	X	X	X	X	X	X	X	X	X	X	X	X
SARS-CoV-2 nucleic acid detection	X								X				X
Vital signs & clinical examination	X[b]	X[b]	X[b]	X[b]	X[b]	X[b]	X[b]	X[b]	X[b]	X[b]	X[b]	X[b]	X[b]
Lab tests	X[c]	X[c]	X[c]	X[c]	X[c]	X[c]	X[c]	X[c]	X[c]	X[c]	X[c]	X[c]	X[c]
ECG	X			X					X		X		X
Chest X-ray	X								X				X
SOFA score	X	X	X	X	X	X	X	X	X	X	X	X	X
Pneumonia Severity Index (PSI)	X	X	X		X		X		X	X	X	X	X
CURB65 score	X	X	X		X		X		X		X	X	X
Adverse events	X	X	X	X	X	X	X	X	X	X	X	X	X

a: Demographics as in WHO Global COVID-19 Clinical Platform.

b: Measurement of temperature, heart rate, respiratory rate, systolic and diastolic blood pressure, oxygen saturation.

c: see Table Laboratory tests.

Table S4. Laboratory tests

Test	Enrolment	Randomization	Treatment										
	Days												
	-1	0	1	2	3	4	5	6	7	10	14	21	28
Angiotensin peptides	X								X				X
Direct renin concentration and plasma aldosterone concentration	X								X				X
Angiotensin-converting enzyme (ACE)	X								X				X
(ACE2	X								X				X
Interleukin 6 (IL-6),	X			X		X			X				X
Soluble tumor necrosis factor receptor type II (sTNFrII)	X			X		X			X				X
Plasminogen activator inhibitor type-1 (PAI-1)	X			X		X			X				X
Tumor necrosis factor-α (TNF-α)	X			X		X			X				X
Soluble receptor for advanced glycation end products (sRAGE) and Surfactant Protein-D	X			X		X			X				X
Endothelin-1	X			X		X			X				X
Lymphocyte and neutrophil counts	X	X	X	X	X	X	X	X	X	X	X	X	X
SARS-Cov2 IgM/IgG***	X	X				X			X		X	X	X
Haemoglobin and red blood cells	X	X						X	X	X	X	X	X
Platelet counts	X	X	X	X	X	X	X	X	X	X	X	X	X
PT-INR, aPTT, fibrinogen, D-Dimer and fibrinogen	X	X	X	X	X	X	X	X	X	X	X	X	X

degradation products (FDP)													
Serum ferritin	X			X		X			X				X
Lactate dehydrogenase	X			X		X			X				X
C-reactive protein and procalcitonin	X			X		X			X				X
S-Urea, creatinine, eGFR	X	X	X	X	X	X	X	X	X	X	X	X	X
S-Na+, S-K+,	X	X	X	X	X	X	X	X	X	X	X	X	X
U-Na+,US-K+,	X	X	X	X	X	X	X	X	X	X	X	X	X
AST ALT	X	X	X	X	X	X	X	X	X	X	X	X	X
High-sensitivity cardiac troponin*	X			X		X			X				X
Viral RNA titer	X			X									
Pregnancy Test**	X												
Urinalysis	X			X		X			X				X

* At any time if patient symptomatic for chest pain or other symptoms suggesting myocardial ischemia

If positive, do not enroll the patient. * These variables will be assessed also after discharge at 3, 6, 12 and 24 months.

Table S5. Secondary Outcomes

Efficacy Outcomes

- Rate of responders

Responder is defined as patient showing improvement of at least two points in seven-category ordinal scale at day 7, 10 ,14, 21 and 28

- Proportion of patients who progressed to critical illness, defined as need of mechanical ventilation, or death
- Change in pO2/FiO2 ratio over time
- Change Sequential organ failure assessment score (SOFA score) over time
- Improvement at imaging, as assessed at the chest CT
- Duration of hospitalization (days) in survivors
- Proportion of patients who require mechanical ventilation and its duration

Safety Outcomes

- Adverse events (fatal and non-fatal) occurring during treatment
- Proportion of premature discontinuation of treatment due to adverse events
- Proportion of patients who develop cardiovascular disease not present at the baseline (*e.g. arrhythmia, myocardial infarction*)
- Hyperkalemia defined as S-$K^+ \geq 5.0$ mmol/L
- Hyponatremia defined as $Na^+ < 130.0$ mmol/L
- Hemorrhages

Safety Biochemical Outcomes

Changes in biomarkers over time

- Coagulation (platelet count, PT-INR, aPTT, fibrinogen, D-Dimer and fibrinogen)
- Infection/inflammation/tissue damage (C-reactive protein, procalcitonin, S-ferritin, AST, ALT)
- Renal function (urea, creatinine, eGFR [calculated as CKD-Epi],S-Na^+ and K^+)

Table S6. Secondary biomarker endpoints

**Coagulation and disseminated intravascular coagulation (DIC)**<br>PT-INR, aPTT, fibrinogen, D-Dimer and fibrinogen degradation products (FDP), factor XII, X, VII and II activity, Plasminogen activator inhibitor type-1 (PAI-1)
**Renin-angiotensin-aldosterone system**<br>Circulating plasma levels of angiotensin peptides (angiotensin 1-8, angiotensin 1-7, angiotensin 1-5), and angiotensin-converting enzyme-1 (ACE-1) and angiotensin-converting enzyme 2 (ACE-2) activity, Direct renin concentration, Plasma aldosterone concentration
**Infection/inflammation/tissue damage**<br>Measurement of viral RNA titer over time and area under-the-curve (AUC)<br>Interleukin 6 (IL-6), Soluble tumor necrosis factor receptor type II (sTNFrII), Tumor necrosis factor-α (TNF-α), Soluble receptor for advanced glycation end products (sRAGE) and Surfactant Protein-D (markers of lung injury),<br>AST, ALT, High-sensitivity cardiac troponin I, C-reactive protein and procalcitonin, serum ferritin, lactate dehydrogenase
**Endothelial dysfunction**<br>C-terminal fragment of endothelin-1, Von Willebrand factor (vWF);
**Blood cells and immunity**<br>Lymphocyte and neutrophil counts,<br>Hemoglobin, red blood cells and platelet count,<br>IgM and IgG for Sars-Cov-2 antigens will be assessed during the in-hospital phase and afterward up to 24 months in order to determine the effect of nafamostat on acquired immunity
**Renal function**<br>Urea, creatinine, eGFR (CKD-Epi),<br>Serum levels of Na^+ and K^+<br>24-h urinary excretion of Na^+ and K^+, and 24h urine albumin excretion (albumin/creatinine, mg/g)

Table S7. Details of the group-sequential design adopted for the study.

Analysis	N events	Z (Efficacy)	Z (Futility)	Nominal P	Alpha spent	HR at bound for Efficacy	HR at bound for Futility	P(cross) if HR=1 for Efficacy or Futility	P(cross) if HR=0.7 for Efficacy
**1**	67 (33%)	2.96	-2.96	0.0015	0.0015	0.4833	2,069	0.0015	0.0660
**2**	133 (67%)	2.09	-2.09	0.0181	0.0172	0.6952	1.438	0.0187	0.4879
**3**	200 (100%	1.71	-1.71	0.0437	0.0313	0.7848	1.274	0.0500	0.8000
**Total**					0.0500				

P(Cross) is the probability of crossing the bound at or before the given analysis under the assumed hazard ratio (HR). P(cross) if HR=0.7 for Futility is <0.001 at each interim.

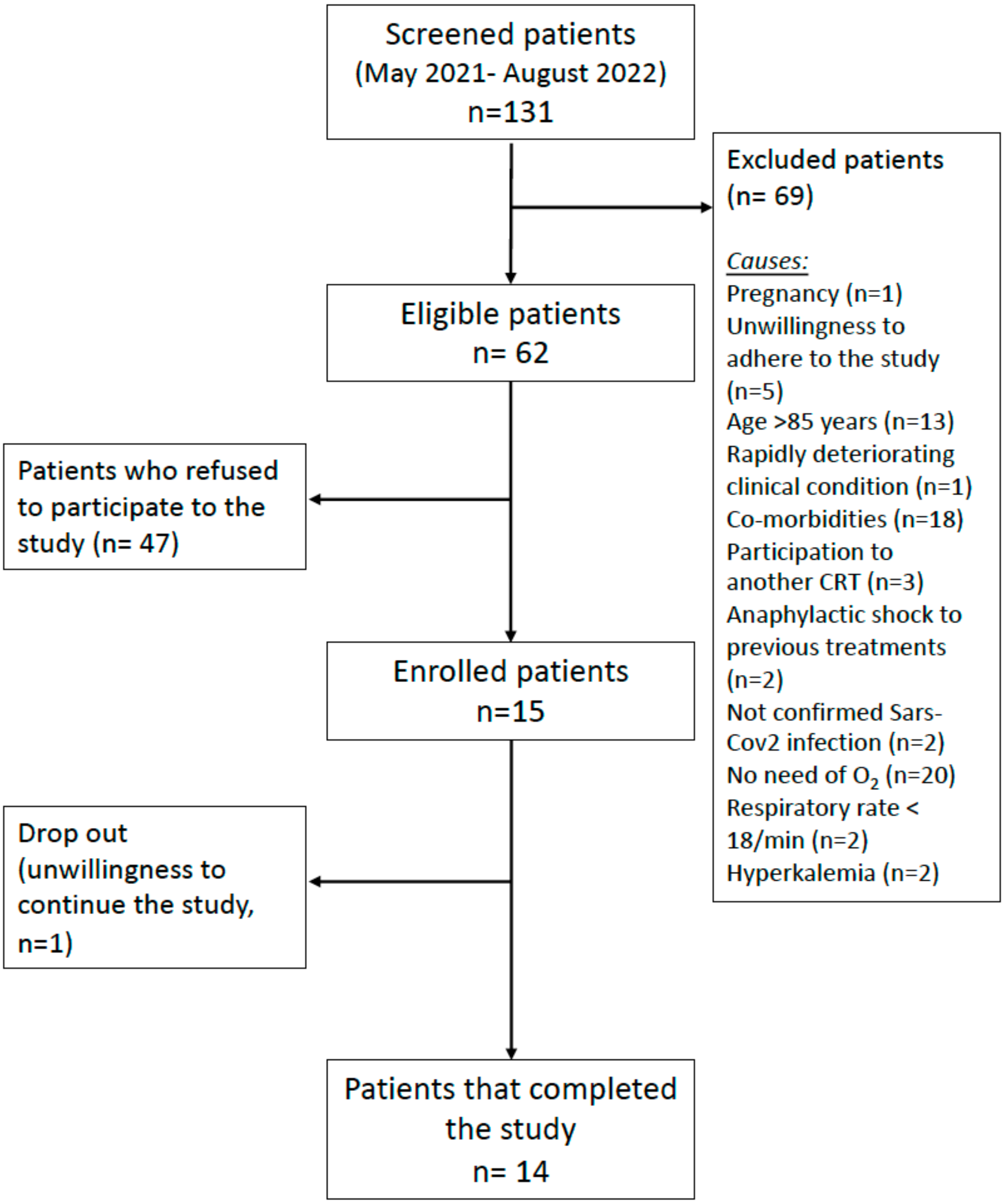

Figure S1. Flow chart of the RACONA Study. After screening patients who were hospitalized because of Covid-19 infection, participation to the RACONA study was offered to those who met inclusion criteria in absence of all causes that precluded their participation (see list of exclusion criteria). One of the 15 enrolled patients, one withdrew the consent from the study and, therefore, the analysis was performed in 14 patients.

ANNEX S1: CONSORT ChecklistCONSORT 2010 checklist of information to include when reporting a randomised trial*

Section/Topic	Item No	Checklist item	Reported on page No
**Title and abstract**			
	1a	Identification as a randomised trial in the title	1
	1b	Structured summary of trial design, methods, results, and conclusions (for specific guidance see CONSORT for abstracts)	1
**Introduction**			
Background and objectives	2a	Scientific background and explanation of rationale	1-2
	2b	Specific objectives or hypotheses	2-3
**Methods**			
Trial design	3a	Description of trial design (such as parallel, factorial) including allocation ratio	3 & Supplemental data
	3b	Important changes to methods after trial commencement (such as eligibility criteria), with reasons	N.A.
Participants	4a	Eligibility criteria for participants	5 & Supplemental data
	4b	Settings and locations where the data were collected	Supplemental data
Interventions	5	The interventions for each group with sufficient details to allow replication, including how and when they were actually administered	3 & Supplemental data
Outcomes	6a	Completely defined pre-specified primary and secondary outcome measures, including how and when they were assessed	2-3 & Supplemental data
	6b	Any changes to trial outcomes after the trial commenced, with reasons	N.A.
Sample size	7a	How sample size was determined	Supplemental data
	7b	When applicable, explanation of any interim analyses and stopping guidelines	4; 10; Supplemental data

Randomisation:			Supplemental data
Sequence generation	8a	Method used to generate the random allocation sequence	
	8b	Type of randomisation; details of any restriction (such as blocking and block size)	Supplemental data
Allocation concealment mechanism	9	Mechanism used to implement the random allocation sequence (such as sequentially numbered containers), describing any steps taken to conceal the sequence until interventions were assigned	Supplemental data
Implementation	10	Who generated the random allocation sequence, who enrolled participants, and who assigned participants to interventions	Supplemental data
Blinding	11a	If done, who was blinded after assignment to interventions (for example, participants, care providers, those assessing outcomes) and how	Supplemental data
	11b	If relevant, description of the similarity of interventions	3 & Supplemental data
Statistical methods	12a	Statistical methods used to compare groups for primary and secondary outcomes	3 & Supplemental data
	12b	Methods for additional analyses, such as subgroup analyses and adjusted analyses	3 & Supplemental data
**Results**			
Participant flow (a diagram is strongly recommended)	13a	For each group, the numbers of participants who were randomly assigned, received intended treatment, and were analysed for the primary outcome	Supplemental data
	13b	For each group, losses and exclusions after randomisation, together with reasons	Supplemental data
Recruitment	14a	Dates defining the periods of recruitment and follow-up	4
	14b	Why the trial ended or was stopped	4,10
Baseline data	15	A table showing baseline demographic and clinical characteristics for each group	4
Numbers analysed	16	For each group, number of participants (denominator) included in each analysis and whether the analysis was by original assigned groups	Supplemental data
Outcomes and estimation	17a	For each primary and secondary outcome, results for each group, and the estimated effect size and its precision (such as 95% confidence interval)	5-9

	17b	For binary outcomes, presentation of both absolute and relative effect sizes is recommended	9
Ancillary analyses	18	Results of any other analyses performed, including subgroup analyses and adjusted analyses, distinguishing pre-specified from exploratory	9
Harms	19	All important harms or unintended effects in each group (for specific guidance see CONSORT for harms)	7
**Discussion15**			
Limitations	20	Trial limitations, addressing sources of potential bias, imprecision, and, if relevant, multiplicity of analyses	15
Generalisability	21	Generalisability (external validity, applicability) of the trial findings	17
Interpretation	22	Interpretation consistent with results, balancing benefits and harms, and considering other relevant evidence	9-11
**Other information**			11
Registration	23	Registration number and name of trial registry	
Protocol	24	Where the full trial protocol can be accessed, if available	Supplemental data; www.clinicaltrial.gov
Funding	25	Sources of funding and other support (such as supply of drugs), role of funders	11

*We strongly recommend reading this statement in conjunction with the CONSORT 2010 Explanation and Elaboration for important clarifications on all the items. If relevant, we also recommend reading CONSORT extensions for cluster randomised trials, non-inferiority and equivalence trials, non-pharmacological treatments, herbal interventions, and pragmatic trials. Additional extensions are forthcoming: for those and for up to date references relevant to this checklist, see www.consort-statement.org.
